# Bis[diamminesilver(I)] 5-nitro­iso­phthalate monohydrate

**DOI:** 10.1107/S1600536810007725

**Published:** 2010-03-13

**Authors:** Di Sun, Geng-Geng Luo, Na Zhang, Rong-Bin Huang

**Affiliations:** a Department of Chemistry, College of Chemistry and Chemical Engineering, Xiamen University, Xiamen 361005, People’s Republic of China

## Abstract

In the title compound, [Ag(NH_3_)_2_]_2_(C_8_H_3_NO_6_)·H_2_O, the cations have an almost linear coordination geometry with two ammine ligands and inter­act with the water mol­ecules [Ag⋯O_water_ = 2.725 (4) and 2.985 (4) Å]. In the crystal, N—H⋯O and O—H⋯O hydrogen bonds, combined with weak (lone pair)⋯π [O⋯centroid distance = 3.401 (4) Å] and π–π stacking [centroid–centroid distance = 3.975 (3) Å] inter­actions, stabilize the three-dimensional supra­molecular network.

## Related literature

For general background to crystal engineering and supra­molecular chemistry, see: Batten & Robson (1998 ▶); Blake *et al.* (1999 ▶); Yaghi *et al.* (2003 ▶). For general background to non-covalent inter­actions, see: Biswas *et al.* (2009 ▶); Egli & Arkhel (2007 ▶); Jeffrey *et al.* (1985 ▶); Mooibroek *et al.* (2006 ▶); Nishio *et al.* (1998 ▶); Rahman *et al.* (2003 ▶). For related structures, see: Sun, Luo, Huang *et al.* (2009 ▶); Sun, Luo, Xu *et al.* (2009 ▶); Sun, Luo, Zhang *et al.* (2009 ▶); You & Zhu (2004 ▶); You *et al.* (2004 ▶); Zheng *et al.* (2002 ▶, 2007 ▶).
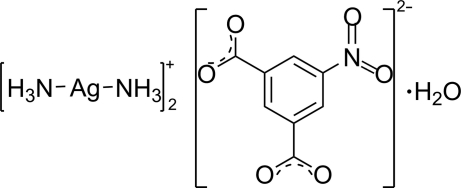


## Experimental

### 

#### Crystal data

[Ag(NH_3_)_2_]_2_(C_8_H_3_NO_6_)·H_2_O
*M* *_r_* = 511.01
Monoclinic, 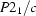

*a* = 7.692 (2) Å
*b* = 12.229 (3) Å
*c* = 16.379 (4) Å
β = 102.100 (4)°
*V* = 1506.5 (7) Å^3^
*Z* = 4
Mo *K*α radiation
μ = 2.64 mm^−1^
*T* = 298 K
0.11 × 0.10 × 0.08 mm

#### Data collection

Oxford Diffraction Gemini S Ultra diffractometer
Absorption correction: multi-scan (*CrysAlis RED*; Oxford Diffraction, 2006 ▶) *T*_min_ = 0.760, *T*_max_ = 0.817
7118 measured reflections
2627 independent reflections
2500 reflections with *I* > 2σ(*I*)
*R*_int_ = 0.036

#### Refinement

*R*[*F* ^2^ > 2σ(*F* ^2^)] = 0.046
*wR*(*F* ^2^) = 0.114
*S* = 1.22
2627 reflections
204 parameters
H-atom parameters constrained
Δρ_max_ = 0.97 e Å^−3^
Δρ_min_ = −0.98 e Å^−3^

### 

Data collection: *CrysAlis CCD* (Oxford Diffraction, 2006 ▶); cell refinement: *CrysAlis RED* (Oxford Diffraction, 2006 ▶); data reduction: *CrysAlis RED*; program(s) used to solve structure: *SHELXS97* (Sheldrick, 2008 ▶); program(s) used to refine structure: *SHELXL97* (Sheldrick, 2008 ▶); molecular graphics: *DIAMOND* (Brandenburg, 1999 ▶) and *SHELXTL* (Sheldrick, 2008 ▶); software used to prepare material for publication: *SHELXL97* and *publCIF* (Westrip, 2010 ▶).

## Supplementary Material

Crystal structure: contains datablocks I, global. DOI: 10.1107/S1600536810007725/hy2282sup1.cif

Structure factors: contains datablocks I. DOI: 10.1107/S1600536810007725/hy2282Isup2.hkl

Additional supplementary materials:  crystallographic information; 3D view; checkCIF report

## Figures and Tables

**Table 1:** Selected bond lengths (Å)

Ag1—N1	2.112 (5)
Ag1—N2	2.105 (5)
Ag2—N3	2.088 (4)
Ag2—N4	2.094 (4)

**Table 2:** Hydrogen-bond geometry (Å, °)

*D*—H⋯*A*	*D*—H	H⋯*A*	*D*⋯*A*	*D*—H⋯*A*
O1*W*—H1*WA*⋯O1^i^	0.85	1.96	2.807 (5)	177
O1*W*—H1*WB*⋯O3	0.85	1.88	2.684 (5)	156
N1—H1*B*⋯O4^ii^	0.89	2.35	3.082 (6)	140
N1—H1*C*⋯O1	0.89	2.10	2.905 (6)	149
N2—H2*A*⋯O3^iii^	0.89	2.09	2.954 (6)	164
N2—H2*B*⋯O4^iv^	0.89	2.36	3.218 (6)	163
N2—H2*C*⋯O1*W*^ii^	0.89	2.28	3.059 (6)	147
N2—H2*C*⋯O6	0.89	2.57	2.990 (6)	110
N3—H3*A*⋯O2^v^	0.89	2.08	2.937 (6)	163
N3—H3*B*⋯O1^iv^	0.89	2.06	2.930 (6)	167
N3—H3*C*⋯O4	0.89	2.02	2.901 (5)	173
N4—H4*A*⋯O4^vi^	0.89	2.23	3.088 (6)	163
N4—H4*B*⋯O3^vii^	0.89	2.14	3.024 (6)	176
N4—H4*C*⋯O2^ii^	0.89	2.15	3.036 (5)	175

Symmetry codes: (i) 
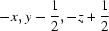
; (ii) 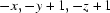; (iii) 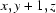; (iv) 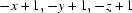; (v) 
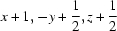
; (vi) 
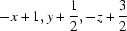
; (vii) 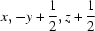.

**Fig. 1.**
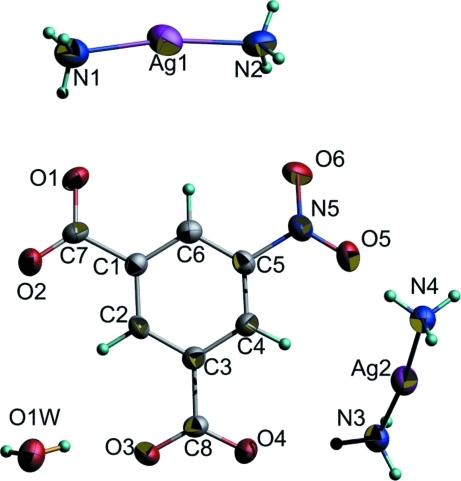
Molecular structure of the title compound, showing the coordination environment around the AgI center. Displacement ellipsoids are drawn at the 50% probability level.

**Fig. 2.**
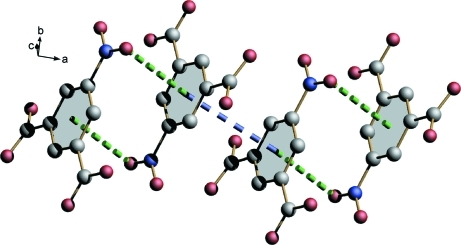
A ball-stick perspective view of the lp(O)···π (green dashed lines) and π–π (blue dashed lines) interactions in the title compound. H atoms and [Ag(NH3)2]+ cations have been omitted for clarity.

**Fig. 3.**
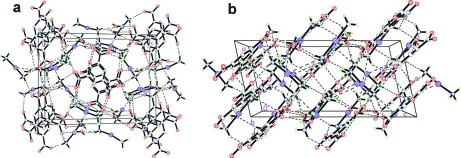
Perspective views of the three-dimensional supramolecular network incorporating N—H···O and O—H···O hydrogen bonds (dashed lines) viewed along two different directions.

## supplementary crystallographic information

### Comment

Renaissance in crystal engineering and supramolecular chemistry is due to the diverse and aesthetic structural topologies and potential use in optical, electrical, catalytic, gas storage and even drug delivery as functional solid materials (Batten & Robson, 1998; Blake *et al.*, 1999; Yaghi *et al.*, 2003). In addition to coordination bonds, noncovalent interactions such as hydrogen bond, π–π stacking, C—H···π, anion···π, cation···π and lone-pair(lp)···π interactions between molecules also play a pivotal role in the stability of molecule packing and govern the physicochemical properties of molecular systems in the condensed phase (Mooibroek *et al.*, 2006; Nishio *et al.*, 1998). Although Ag^I^ ion under ammoniacal conditions can form {[Ag(NH_3_)_2_]^+^}_n_ (n = 1 or 2) (Zheng *et al.*, 2007), which can be stabilized by supramolecular interactions, only limited [Ag(NH_3_)_2_]-containing compounds were documented due to the weak Ag—N_ammine_ coordination bond (You *et al.*, 2004; Zheng *et al.*, 2002). Recently, we have pursued systematic investigations about the self-assembly of Ag^I^ ion with different bipodal *N*-donor ligands and multicarboxylates under ammoniacal conditions (Sun, Luo, Huang *et al.*, 2009; Sun, Luo, Xu *et al.*, 2009; Sun, Luo, Zhang *et al.*, 2009). In an attempt to exploit the Ag^I^/aminopyrazine/H_2_nipa system (H_2_nipa = 5-nitroisophthalic acid), we surprisingly obtained the title compound.

The title compound comprises two [Ag(NH_3_)_2_]^+^ cations, one nipa anion and one uncoordinated water molecule in the asymmetric unit (Fig. 1). Each Ag^I^ ion is in an almost linear coordination environment, coordinated by two ammonia molecules, forming a cationic [Ag(NH_3_)_2_]^+^ monomer. The Ag—N bond lengths range from 2.088 (4) to 2.112 (5) Å (Table 1), which are comparable to the corresponding values observed in other silver(I) compounds (You & Zhu, 2004). The N1—Ag1—N2 [169.90 (19)°] and N3—Ag2—N4 [174.05 (16)°] angles deviate from the ideal 180°, as a result of weak interactions between the Ag^I^ ions and water molecules. The Ag1···O1W^iii^ and Ag2···O1W^v^ distances are 2.725 (4) and 2.985 (4) Å, respectively, which suggest anything other than a secondary interaction [symmetry codes: (iii) x, y+1, z; (v) x+1, -y+1/2, z+1/2]. The shortest centroid–centroid distance between neighboring phenyl rings of nipa anions is 3.975 (3) Å, with a large slippage of 2.129 Å, which suggests the existence of a weak offset π–π stacking interaction. On the other hand, one striking feature of the title compound is an lp···π interaction (Biswas *et al.*, 2009; Egli & Arkhel, 2007). A weak lp···π interaction is observed between the nitro O5 atom and phenyl ring of the nipa anion. The distance between the ring centroid and O5 atom is 3.401 (4) Å. This lp(O)···π interaction distance falls in the range of few experimental examples so far reported (Rahman *et al.*, 2003). The angle θ (which corresponds to the angle between the O atom, the ring centroid and the aromatic plane) is 83.7 (3)°, reflecting a significant lp···π interaction. Every two nipa anions arrange in a parallel manner, forming a dimer through lp(O)···π interactions, and the neighboring dimers pack togther through weak π–π stacking interactions into columns running along the *a* axis (Fig. 2).

One of the ammonia molecules forms a bifurcated hydrogen bond (Jeffrey *et al.*, 1985) [N2–H2C···O1W^ii^ and N2–H2C···O6, symmetry code: (ii) -x, -y+1, -z+1]. In addition, the [Ag(NH_3_)_2_]^+^ cations, nipa anions and water molecules interact *via* N—H···O and O—H···O hydrogen bonds (Table 2) [average N···O = 3.010 (6), O···O = 2.746 (5) Å] to consolidate the three-dimensional supramolecular network (Fig. 3).

### Experimental

All reagents and solvents were used as obtained commercially without further purification. A mixture of Ag_2_O (116 mg, 0.5 mmol), 2-aminopyrazine (95 mg, 1 mmol) and H_2_nipa (211 mg, 1 mmol) were stirred in CH_3_CN/H_2_O mixed solvent (8 ml, v/v = 1:1). Then aqueous NH_3_ solution (25%) was dropped into the mixture to give a clear solution under ultrasonic treatment. The resultant solution was allowed to evaporate slowly in darkness at room temperature for several days to give colorless crystals of the title compound (yield 61%). They were washed with a small volume of cold CH_3_CN and diethyl ether. Analysis calculated for C_8_H_17_Ag_2_N_5_O_7_: C 18.80, H 3.35, N 13.71%; found: C 18.86, H 3.39, N 13.64%.

### Refinement

C- and N-bound H atoms were placed in calculated positions and refined using a riding model, with C—H = 0.93 and N—H = 0.89 Å and with *U*_iso_(H) = 1.2*U*_eq_(C,N). H atoms of water molecule were located in a difference Fourier map and refined as riding, with O—H = 0.85 Å and *U*_iso_(H) = 1.2*U*_eq_(O).

### Crystal data

**Table:**

[Ag(NH_3_)_2_]_2_(C_8_H_3_NO_6_)·H_2_O	*F*(000) = 1000
*M**_r_* = 511.01	*D*_x_ = 2.253 Mg m^−^^3^
Monoclinic, *P*2_1_/*c*	Mo *K*α radiation, λ = 0.71073 Å
Hall symbol: -P 2ybc	Cell parameters from 4729 reflections
*a* = 7.692 (2) Å	θ = 5.1–57.2°
*b* = 12.229 (3) Å	µ = 2.64 mm^−^^1^
*c* = 16.379 (4) Å	*T* = 298 K
β = 102.100 (4)°	Block, colorless
*V* = 1506.5 (7) Å^3^	0.11 × 0.10 × 0.08 mm
*Z* = 4	

### Data collection

**Table:**

Oxford Diffraction Gemini S Ultra diffractometer	2627 independent reflections
Radiation source: sealed tube	2500 reflections with *I* > 2σ(*I*)
graphite	*R*_int_ = 0.036
Detector resolution: 16.1903 pixels mm^-1^	θ_max_ = 25.0°, θ_min_ = 2.1°
ω scans	*h* = −9→9
Absorption correction: multi-scan (*CrysAlis RED*; Oxford Diffraction, 2006)	*k* = −8→14
*T*_min_ = 0.760, *T*_max_ = 0.817	*l* = −17→19
7118 measured reflections	

### Refinement

**Table:**

Refinement on *F*^2^	Secondary atom site location: difference Fourier map
Least-squares matrix: full	Hydrogen site location: inferred from neighbouring sites
*R*[*F*^2^ > 2σ(*F*^2^)] = 0.046	H-atom parameters constrained
*wR*(*F*^2^) = 0.114	*w* = 1/[σ^2^(*F*_o_^2^) + (0.0451*P*)^2^ + 2.1461*P*] where *P* = (*F*_o_^2^ + 2*F*_c_^2^)/3
*S* = 1.22	(Δ/σ)_max_ < 0.001
2627 reflections	Δρ_max_ = 0.97 e Å^−^^3^
204 parameters	Δρ_min_ = −0.98 e Å^−^^3^
0 restraints	Extinction correction: *SHELXL97* (Sheldrick, 2008), Fc^*^=kFc[1+0.001xFc^2^λ^3^/sin(2θ)]^-1/4^
Primary atom site location: structure-invariant direct methods	Extinction coefficient: 0.0264 (13)

### Fractional atomic coordinates and isotropic or equivalent isotropic displacement parameters (Å^2^)

**Table:**

	*x*	*y*	*z*	*U*_iso_*/*U*_eq_	
Ag1	0.01233 (6)	0.87015 (4)	0.39354 (3)	0.0561 (2)	
Ag2	0.56988 (5)	0.37352 (3)	0.78293 (3)	0.0444 (2)	
C1	0.1106 (5)	0.4687 (4)	0.4096 (3)	0.0272 (9)	
C2	0.1513 (6)	0.3606 (4)	0.4283 (3)	0.0277 (9)	
H2	0.0955	0.3065	0.3922	0.033*	
C3	0.2729 (5)	0.3307 (4)	0.4993 (3)	0.0273 (9)	
C4	0.3475 (6)	0.4110 (4)	0.5543 (3)	0.0294 (10)	
H4	0.4278	0.3929	0.6032	0.035*	
C5	0.3022 (6)	0.5176 (4)	0.5362 (3)	0.0281 (9)	
C6	0.1878 (6)	0.5481 (4)	0.4639 (3)	0.0294 (10)	
H6	0.1631	0.6216	0.4521	0.035*	
C7	−0.0153 (6)	0.4982 (4)	0.3292 (3)	0.0308 (10)	
C8	0.3273 (6)	0.2131 (4)	0.5152 (3)	0.0293 (10)	
N1	−0.1928 (7)	0.8046 (4)	0.3002 (3)	0.0544 (12)	
H1A	−0.1867	0.8333	0.2509	0.065*	
H1B	−0.2977	0.8205	0.3120	0.065*	
H1C	−0.1805	0.7323	0.2983	0.065*	
N2	0.2262 (6)	0.9066 (4)	0.4929 (3)	0.0499 (11)	
H2A	0.2441	0.9786	0.4954	0.060*	
H2B	0.3239	0.8733	0.4846	0.060*	
H2C	0.2011	0.8835	0.5406	0.060*	
N3	0.7101 (6)	0.2725 (4)	0.7167 (3)	0.0411 (10)	
H3A	0.7576	0.2171	0.7490	0.049*	
H3B	0.7962	0.3108	0.7012	0.049*	
H3C	0.6364	0.2466	0.6716	0.049*	
N4	0.4096 (5)	0.4767 (4)	0.8378 (2)	0.0395 (10)	
H4A	0.4737	0.5333	0.8615	0.047*	
H4B	0.3677	0.4399	0.8764	0.047*	
H4C	0.3192	0.5008	0.7988	0.047*	
N5	0.3862 (6)	0.6021 (4)	0.5942 (2)	0.0358 (9)	
O1	−0.0137 (5)	0.5948 (3)	0.3058 (2)	0.0405 (8)	
O1W	−0.0469 (5)	0.0861 (3)	0.3572 (2)	0.0502 (9)	
H1WA	−0.0315	0.0871	0.3073	0.060*	
H1WB	0.0474	0.1189	0.3813	0.060*	
O2	−0.1083 (5)	0.4259 (3)	0.2916 (2)	0.0502 (10)	
O3	0.2508 (5)	0.1444 (3)	0.4657 (3)	0.0494 (10)	
O4	0.4452 (5)	0.1927 (3)	0.5764 (2)	0.0412 (8)	
O5	0.4957 (5)	0.5735 (3)	0.6553 (2)	0.0509 (10)	
O6	0.3436 (6)	0.6952 (3)	0.5786 (2)	0.0567 (11)	

### Atomic displacement parameters (Å^2^)

**Table:**

	*U*^11^	*U*^22^	*U*^33^	*U*^12^	*U*^13^	*U*^23^
Ag1	0.0498 (3)	0.0475 (3)	0.0734 (4)	−0.00206 (19)	0.0184 (2)	−0.0061 (2)
Ag2	0.0446 (3)	0.0460 (3)	0.0402 (3)	0.00187 (17)	0.00340 (18)	−0.00385 (17)
C1	0.027 (2)	0.030 (2)	0.025 (2)	0.0008 (18)	0.0062 (16)	0.0021 (18)
C2	0.032 (2)	0.026 (2)	0.024 (2)	−0.0037 (18)	0.0049 (17)	−0.0045 (17)
C3	0.024 (2)	0.028 (2)	0.030 (2)	0.0005 (18)	0.0057 (17)	0.0014 (19)
C4	0.030 (2)	0.031 (2)	0.027 (2)	0.0013 (19)	0.0049 (17)	0.0026 (19)
C5	0.030 (2)	0.028 (2)	0.027 (2)	−0.0044 (19)	0.0088 (17)	−0.0030 (18)
C6	0.028 (2)	0.029 (2)	0.032 (2)	0.0017 (19)	0.0104 (18)	0.0025 (19)
C7	0.030 (2)	0.028 (3)	0.034 (2)	0.005 (2)	0.0069 (18)	0.006 (2)
C8	0.031 (2)	0.027 (2)	0.031 (2)	0.0054 (19)	0.0082 (19)	0.0019 (19)
N1	0.061 (3)	0.048 (3)	0.055 (3)	0.006 (2)	0.016 (2)	0.001 (2)
N2	0.052 (3)	0.038 (3)	0.062 (3)	0.006 (2)	0.015 (2)	0.009 (2)
N3	0.038 (2)	0.038 (2)	0.043 (2)	−0.0028 (19)	−0.0014 (18)	−0.0036 (19)
N4	0.039 (2)	0.039 (2)	0.039 (2)	−0.0006 (19)	0.0032 (17)	0.0000 (19)
N5	0.042 (2)	0.035 (2)	0.031 (2)	−0.0080 (19)	0.0082 (18)	−0.0020 (18)
O1	0.044 (2)	0.0333 (19)	0.0391 (19)	0.0038 (16)	−0.0021 (15)	0.0113 (16)
O1W	0.045 (2)	0.059 (3)	0.043 (2)	0.0005 (19)	0.0004 (16)	−0.0007 (19)
O2	0.054 (2)	0.037 (2)	0.047 (2)	−0.0080 (18)	−0.0167 (17)	0.0017 (18)
O3	0.060 (2)	0.0287 (19)	0.049 (2)	0.0045 (17)	−0.0119 (18)	−0.0091 (17)
O4	0.0412 (18)	0.0344 (19)	0.0393 (18)	0.0044 (15)	−0.0112 (15)	0.0000 (15)
O5	0.060 (2)	0.046 (2)	0.039 (2)	−0.0077 (19)	−0.0089 (17)	−0.0042 (17)
O6	0.088 (3)	0.027 (2)	0.049 (2)	0.002 (2)	−0.001 (2)	−0.0022 (17)

### Geometric parameters (Å, °)

**Table:**

Ag1—N1	2.112 (5)	C7—O1	1.243 (6)
Ag1—N2	2.105 (5)	C8—O3	1.228 (6)
Ag2—N3	2.088 (4)	C8—O4	1.229 (5)
Ag2—N4	2.094 (4)	N1—H1A	0.8900
Ag1—O1W^i^	2.725 (4)	N1—H1B	0.8900
Ag2—O1W^ii^	2.985 (4)	N1—H1C	0.8900
C1—C6	1.367 (6)	N2—H2A	0.8900
C1—C2	1.378 (6)	N2—H2B	0.8900
C1—C7	1.505 (6)	N2—H2C	0.8900
C2—C3	1.380 (6)	N3—H3A	0.8900
C2—H2	0.9300	N3—H3B	0.8900
C3—C4	1.374 (6)	N3—H3C	0.8900
C3—C8	1.505 (6)	N4—H4A	0.8900
C4—C5	1.366 (6)	N4—H4B	0.8900
C4—H4	0.9300	N4—H4C	0.8900
C5—C6	1.370 (6)	N5—O6	1.198 (6)
C5—N5	1.460 (6)	N5—O5	1.217 (5)
C6—H6	0.9300	O1W—H1WA	0.8501
C7—O2	1.220 (6)	O1W—H1WB	0.8500
			
N2—Ag1—N1	169.90 (19)	O4—C8—C3	117.8 (4)
N3—Ag2—N4	174.05 (16)	Ag1—N1—H1A	109.5
N2—Ag1—O1W^i^	91.79 (15)	Ag1—N1—H1B	109.5
N1—Ag1—O1W^i^	98.31 (16)	H1A—N1—H1B	109.5
N3—Ag2—O1W^ii^	74.69 (14)	Ag1—N1—H1C	109.5
N4—Ag2—O1W^ii^	110.21 (14)	H1A—N1—H1C	109.5
C6—C1—C2	119.3 (4)	H1B—N1—H1C	109.5
C6—C1—C7	120.7 (4)	Ag1—N2—H2A	109.5
C2—C1—C7	119.9 (4)	Ag1—N2—H2B	109.5
C1—C2—C3	121.6 (4)	H2A—N2—H2B	109.5
C1—C2—H2	119.2	Ag1—N2—H2C	109.5
C3—C2—H2	119.2	H2A—N2—H2C	109.5
C4—C3—C2	118.7 (4)	H2B—N2—H2C	109.5
C4—C3—C8	120.4 (4)	Ag2—N3—H3A	109.5
C2—C3—C8	120.9 (4)	Ag2—N3—H3B	109.5
C5—C4—C3	119.1 (4)	H3A—N3—H3B	109.5
C5—C4—H4	120.4	Ag2—N3—H3C	109.5
C3—C4—H4	120.4	H3A—N3—H3C	109.5
C4—C5—C6	122.4 (4)	H3B—N3—H3C	109.5
C4—C5—N5	118.5 (4)	Ag2—N4—H4A	109.5
C6—C5—N5	119.0 (4)	Ag2—N4—H4B	109.5
C1—C6—C5	118.8 (4)	H4A—N4—H4B	109.5
C1—C6—H6	120.6	Ag2—N4—H4C	109.5
C5—C6—H6	120.6	H4A—N4—H4C	109.5
O2—C7—O1	125.1 (4)	H4B—N4—H4C	109.5
O2—C7—C1	118.0 (4)	O6—N5—O5	124.1 (4)
O1—C7—C1	116.8 (4)	O6—N5—C5	118.1 (4)
O3—C8—O4	124.7 (4)	O5—N5—C5	117.8 (4)
O3—C8—C3	117.5 (4)	H1WA—O1W—H1WB	99.3

Symmetry codes: (i) *x*, *y*+1, *z*; (ii) *x*+1, −*y*+1/2, *z*+1/2.

### Hydrogen-bond geometry (Å, °)

**Table:**

*D*—H···*A*	*D*—H	H···*A*	*D*···*A*	*D*—H···*A*
O1W—H1WA···O1^iii^	0.85	1.96	2.807 (5)	177
O1W—H1WB···O3	0.85	1.88	2.684 (5)	156
N1—H1B···O4^iv^	0.89	2.35	3.082 (6)	140
N1—H1C···O1	0.89	2.10	2.905 (6)	149
N2—H2A···O3^i^	0.89	2.09	2.954 (6)	164
N2—H2B···O4^v^	0.89	2.36	3.218 (6)	163
N2—H2C···O1W^iv^	0.89	2.28	3.059 (6)	147
N2—H2C···O6	0.89	2.57	2.990 (6)	110
N3—H3A···O2^ii^	0.89	2.08	2.937 (6)	163
N3—H3B···O1^v^	0.89	2.06	2.930 (6)	167
N3—H3C···O4	0.89	2.02	2.901 (5)	173
N4—H4A···O4^vi^	0.89	2.23	3.088 (6)	163
N4—H4B···O3^vii^	0.89	2.14	3.024 (6)	176
N4—H4C···O2^iv^	0.89	2.15	3.036 (5)	175

Symmetry codes: (iii) −*x*, *y*−1/2, −*z*+1/2; (iv) −*x*, −*y*+1, −*z*+1; (i) *x*, *y*+1, *z*; (v) −*x*+1, −*y*+1, −*z*+1; (ii) *x*+1, −*y*+1/2, *z*+1/2; (vi) −*x*+1, *y*+1/2, −*z*+3/2; (vii) *x*, −*y*+1/2, *z*+1/2.
